# Endometrial Cancer Staging: Is There Value in ADC?

**DOI:** 10.3390/jpm13050728

**Published:** 2023-04-25

**Authors:** Ana Sofia Linhares Moreira, Vera Ribeiro, Giacomo Aringhieri, Salvatore Claudio Fanni, Lorenzo Tumminello, Lorenzo Faggioni, Dania Cioni, Emanuele Neri

**Affiliations:** 1 Radiology Department, Centro Hospitalar Universitário do Algarve, 8000-386 Faro, Portugal; 2 Gynaecology Department, Centro Hospitalar Universitário do Algarve, 8000-386 Faro, Portugal; 3 Academic Radiology, Department of Translational Research, University of Pisa, 56126 Pisa, Italy

**Keywords:** endometrial cancer, magnetic resonance imaging, diffusion-weighted imaging, apparent diffusion coefficient, dynamic contrast-enhanced magnetic resonance imaging, staging, grading

## Abstract

Purpose: To assess the ability of apparent diffusion coefficient (ADC) measurements in predicting the histological grade of endometrial cancer. A secondary goal was to assess the agreement between MRI and surgical staging as an accurate measurement. Methods: Patients with endometrial cancers diagnosed between 2018–2020 and having received both MRI and surgical staging were retrospectively enrolled. Patients were characterized according to histology, tumor size, FIGO stage (MRI and surgical stage), and functional MRI parameters (DCE and DWI/ADC). Statistical analysis was performed to determine if an association could be identified between ADC variables and histology grade. Secondarily, we assessed the degree of agreement between the MRI and surgical stages according to the FIGO classification. Results: The cohort included 45 women with endometrial cancer. Quantitative analysis of ADC variables did not find a statistically significant association with histological tumor grades. DCE showed higher sensitivity than DWI/ADC in the assessment of myometrial invasion (85.00% versus 65.00%) with the same specificity (80.00%). A good agreement between MRI and histopathology for the FIGO stage was found (kappa of 0.72, *p* < 0.01). Differences in staging between MRI and surgery were detected in eight cases, which could not be justified by the interval between MRI and surgery. Conclusions. ADC values were not useful for predicting endometrial cancer grade, despite the good agreement between MRI interpretation and histopathology of endometrial cancer staging at our center.

## 1. Introduction

Endometrial carcinoma is the most frequent female genital malignancy in industrialized countries and the second most frequent worldwide after cervical carcinoma [1]. According to the GLOBOCAN cancer statistics, endometrial cancer was the fourth leading cause of death due to gynecological cancer worldwide in 2018 [2]. The age range with the highest incidence of endometrial cancer is between 55 and 64 years (median 62 years) [3].

Many factors have been described to be associated with an increased risk of this disease, such as alcohol consumption, obesity, excess exogenous estrogen, and insulin resistance [4,5,6]. Endometrial carcinoma has also associations with multiple genetic factors, among which PTEN, tumor protein 53, and microsatellite instability of MLH1, MSH2, or MSH6 [6]. Additionally, endometrial cancers may also occur in women with autosomal dominant hereditary syndromes such as Lynch or Cowden syndrome, as well as in carriers of BRCA1 mutations [7,8,9].

The prognosis of endometrial cancer takes into account tumor histological type, tumor grade, International Federation of Gynecology and Obstetrics (FIGO) stage at diagnosis, in particular the depth of myometrial invasion, and molecular markers such as serum CA-125, estrogen and progesterone receptors and microsatellite instability [10,11]. Tumor type and tumor grade are determined histologically and are used as surrogates of tumor aggressiveness, which influences prognosis and treatment [11,12].

Magnetic resonance imaging (MRI) is used as a non-invasive assessment tool of the extent of disease of several gynecological neoplasms, including endometrial carcinoma [13,14,15,16], because of an improved tissue resolution. Both image and surgical staging follow the FIGO classification, with the most determining findings for the management of endometrial cancer being the depth of myometrial invasion, the invasion of the cervix, and the presence and location of lymphadenopathy [12,13,14]. The depth of myometrial invasion is used as a surrogate marker for the presence of lymph node metastases, which are associated with a poorer prognosis. As such, the depth of myometrial invasion has therapeutical implications since stage IA disease (invasion of the myometrium of less than 50%) is treated with hysterectomy, not requiring pelvic lymphadenectomy, while in stage IB disease (invasion more than or equal to 50% of the myometrium, also called deep myometrial infiltration) hysterectomy and pelvic lymphadenectomy are recommended [11,12,14,16].

The ability of MRI to determine the depth of myometrial invasion has extensively been researched in recent years [14]. It is important to precisely quantify through MRI the depth of myometrial invasion (<50% or ≥50%) to avoid unnecessary lymphadenectomy, which may be associated with complications such as massive bleeding, nerve injury, lower extremity lymphedema, and pelvic lymphocele [17].

Recently, functional MRI sequences have been investigated as potentially useful imaging biomarkers in the oncological setting, namely for gynecological cancer imaging [18]. The most frequently used functional sequences in MRI are dynamic contrast enhancement MRI (DCE-MRI) and diffusion-weighted imaging (DWI) with apparent diffusion coefficient maps (ADC) calculation, reflecting vascularity and tissue cellularity, respectively. These sequences are already in use in current clinical practice providing predominantly qualitative information, although quantitative data can also be extracted, in particular from ADC maps [13,14].

ADC maps’ quantitative information is usually derived after volumetric segmentation of the whole tumor, which is effective but also time-consuming and impractical for everyday clinical use [19]. We conceived that the placement of a region of interest (ROI) in the most representative slice of the ADC map would simplify the retrieval of this information and make it more practical for routine clinical implementation.

It is essential to assess the degree of agreement between MRI staging and surgical staging, defined as the gold standard in clinical practice, to determine the accuracy of the imaging diagnosis due to the aforementioned therapeutic implications since most endometrial cancers are diagnosed at an early stage.

The main purpose of this study was to determine if the quantitative information derived from ADC maps using ROI in a representative slice correlates with the histological grade of endometrial cancer, leading to a non-invasive assessment of tumor aggressiveness that would be simple to implement into clinical practice. As a secondary endpoint, the agreement between MRI and pathological staging was investigated.

## 2. Materials and Methods

This was a retrospective, institutional review board-approved (UAIF 164/2021), single-center study of endometrial cancers diagnosed at Centro Hospitalar Universitário do Algarve between 2018 and 2020.

### 2.1. Patient Selection

We assessed all patients with endometrial cancer diagnosed at Centro Hospitalar Universitário do Algarve between 1 January 2018 and 31 December 2020. The inclusion criteria were as follows: having performed a preoperative staging MRI followed by surgery with histopathology analysis of the surgical specimen at our center. The exclusion criteria were the presence of severe artifacts in the MRI scans, the absence of surgical treatment at our center, or having performed neoadjuvant treatments before surgery.

Patient records were analyzed to determine age at initial diagnosis, date and type of surgical treatment (hysterectomy with or without lymphadenectomy), tumor histology, tumor grade, and FIGO surgical stage.

### 2.2. MR Imaging

All studies were performed on a 1.5 Tesla MRI scanner (Magnetom Symphony, Siemens Healthineers, Erlangen, Germany). Bowel peristalsis was decreased in all patients by intravenous injection of 20 mg butylscopolamine bromide (Buscopan, Boehringer, Ingelheim, Ingelheim am Rhein, Germany) immediately before the start of the examination. DCE-MRI was performed before and after contrast agent administration (0.1 mmol gadolinium/kg body weight, Gadovist, Bayer, Germany) with multiple sequential acquisitions starting 30 s after contrast injection. The imaging protocol follows the recommendations of the European Society of Urogenital Radiology guidelines [15], and the acquisition parameters used for the studies are available as Supplementary Material (Table S1).

### 2.3. Image Interpretation

All MRIs were retrospectively reviewed by a single dedicated urogenital radiologist (experience of more than 300 gynecologic MRI scans evaluated), who was blinded to the histopathology reports.

The following data was collected from the MRI analysis: date of examination, lesion volume (determined manually with the ellipsoid formula), staging information (myometrial invasion <50% or ≥50%, cervical invasion, pelvic or lombo-aortic lymphadenopathy), quantification of ADC in the lesion and comparison between DWI and DCE sequences.

**Figure 1:**
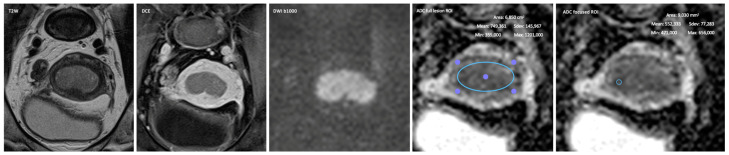
Sixty-year-old female staged at MRI as T1a and proven at pathology as T1a. Depiction of the method used for the quantitative data collection with the two different ROI—full lesion ROI (large circle) and focused ROI (small circle).

**Figure 2:**
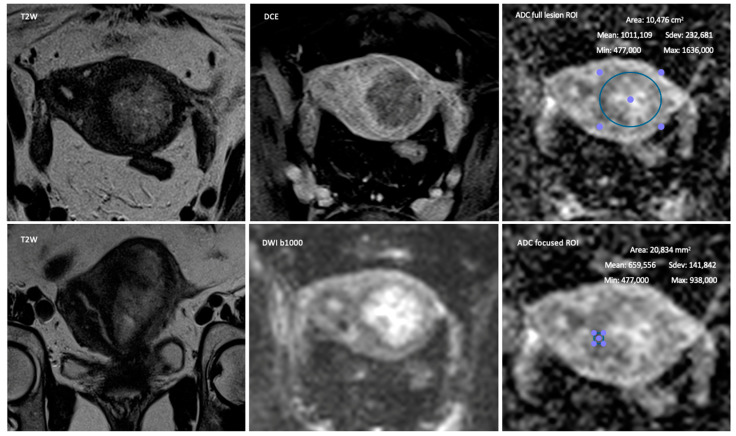
Fifty-seven-year-old female with a Mullerian malformation (septate uterus) staged at MRI as T1b and proven at pathology as T1b. The presence of a uterine malformation is a pitfall for the detection of myometrial infiltration by the tumor. Depiction of the method used for the quantitative data collection with the two different ROI.

**Figure 3:**
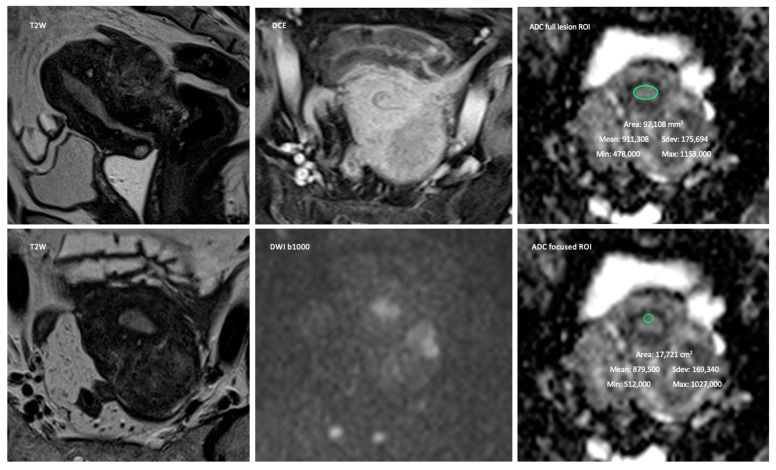
Seventy-year-old female with a leiomyoma staged at MRI as T1a and proven at pathology to be a T1a. Leiomyomas are one of the pitfalls for the determination of myometrial invasion by tumor. Depiction of the method used for the quantitative data collection using the two different ROI.

For the quantification of ADC, two different methods were analyzed, both using regions of interest (ROI) per lesion in a single representative axial oblique slice of the ADC map, one assessing the whole endometrial lesion (full lesion ROI) and another assessing the area with the highest diffusivity-restriction (focused ROI) (Figure 1, Figure 2 and Figure 3).

From each ROI, the mean ADC, minimum ADC, and maximum ADC values were collected.

A direct visual comparison between DWI and DCE sequences was performed to assess the depth of myometrial invasion.

### 2.4. Statistical Analysis

Clinical variables were described with the use of frequencies and percentages for categorical variables. Mean and standard deviation or median and interquartile range were reported for quantitative variables, as appropriate. The Shapiro–Wilk test was used to assess the normality of distribution for quantitative variables.

The differences between subgroups were evaluated with the Chi-square test for categorical variables and by the Mann–Whitney U tests and Kruskal–Wallis H tests for quantitative variables, as appropriate.

The association between the disease grade (G1, G2, and G3) and quantitative ADC variables extracted both from full lesion ROI and focused ROI was investigated.

Secondly, a direct comparison was made between the performance of DCE-MRI and DWI/ADC for the assessment of myometrial invasion depth.

Weighted Cohen’s kappa coefficient was estimated for the analysis of concordance between radiological and pathological staging. For the interpretation of weighted Cohen’s kappa, the following stratification was used: poor agreement or less than chance (k < 0), slight agreement (k 0.01–0.20), fair agreement (k 0.21–0.40), moderate agreement (k 0.41–0.60), good agreement (k 0.61–0.80) and very good agreement (k 0.81–0.99). Subsequently, in discordant cases, the time lag between MRI and surgery was analyzed.

*p* values of less than 0.05 were considered to indicate statistical significance. All statistical analyses were performed with IBM SPSS Statistics for Windows, version 26.0 (IBM Corp., Armonk, NY, USA).

## 3. Results

During the study period, fifty patients received MRIs for endometrial cancer staging. Five patients were excluded due to the lack of surgical treatment. The characteristics of the final cohort of forty-five patients are shown in Table 1.

The majority of our population was diagnosed with endometrioid endometrial cancer (39/45 patients, 86.67%) with a low FIGO stage (MRI stage lower than T2 was found in 86.67% of patients).

The most aggressive tumor histology types in our cohort (serous and undifferentiated carcinomas) were found in 6/45 cases (13.33%). These patients’ initial staging was heterogeneous (two T1a, two T1b, one T3, and one T4).

No statistically significant association was found between the functional ADC variables and the histological tumor grades (Table 2); even when subgroup analysis was performed (G1 versus G2–G3 and G1–G2 versus G3), no statistically significant association was detected.

Comparison between DWI/ADC and DCE for assessing myometrial invasion showed an agreement in 18 cases for invasion <50% (100.00%), an agreement in 23 cases with invasion ≥50% (85.19%) and a discrepancy in 4 cases (14.81%) with DWI suggesting ≥50% invasion and DCE-MRI <50% invasion. In the 4 discordant cases, histopathology depicted an invasion <50%, which was concordant with the DCE assessment for all of the cases. Analysis focusing on DCE for the identification of myometrial depth of infiltration showed a sensitivity of 85.00% and a specificity of 80.00%, with a 100.00% accuracy, while DWI/ADC had a sensitivity of 65.00% with similar specificity (80.00%).

The analysis between tumor grade and depth of myometrial invasion did not identify a statistically significant association (χ^2^ (2,45) = 1.72, *p* = 0.42).

A good agreement between MRI and pathologic stage was found, with 37/45 cases in agreement (82.22%) (Table 3), with a weighted Cohen’s kappa of 0.75 (*p* < 0.01).

As demonstrated in Table 3, the FIGO stage was overestimated in 2/45 cases (4.44%) and underestimated in 6/45 cases (13.33%).

In these 8/45 cases (17.78%) of a discrepancy between the MRI and the surgical staging, we analyzed if there had been a time lag between MRI and surgery that could justify the discrepancies in staging, but no statistical difference was identified (U = 130, *p* = 0.61).

Invasion of the cervix by endometrial cancer was reported at histopathology in 7/45 cases, 3 of which were correctly detected by MRI. MRI had a sensitivity of 42.86% for the detection of cervical invasion in this series, with an accuracy of 91.11%. There were no false positive results of cervical invasion.

Lymphadenectomy was performed in 15/45 patients in our cohort (33.33%), with only 3 cases having positive nodes on histopathological examination. MRI had a 66.67% sensitivity, 91.67% specificity, and 86.67% accuracy for the detection of nodal metastases in our cohort.

A trend of larger lesions presenting in higher-grade tumors was suggested (Table 4), even though this association also did not reach statistical significance (H(2) = 3.26, *p* = 0.20).

No statistically significant difference was found between the tumor grade and lesion volume (*p* = 0.20), even despite a trend depicting larger lesions having a higher tumor grade.

## 4. Discussion

This study investigated the association between quantitative information derived from ADC maps and the histological grade of endometrial cancers using a simplified method of collecting the quantitative data from the ADC maps by using ROI measurements. As a secondary object, the concordance between MRI and pathological staging was investigated.

The characteristics of our population were representative of clinical practice, with the majority having endometrioid endometrial cancer diagnosed at an early FIGO stage. These early cases are the ones that benefit more from the ability of MRI to distinguish the depth of myometrial infiltration and have more clinical impact in determining the type of surgery that should be performed [11,12,14,16].

ADC quantitative variables were extracted using two different-sized ROIs to consider for whole tumor heterogeneity by using the full lesion ROI and to more precisely depict tumor aggressiveness by using the focused ROI in the area with the highest diffusivity-restriction. Nevertheless, the different ADC values derived did not show any statistically significant correlation with tumor grade for any of the analyzed variables (Table 2), as previously reported in the literature [20,21,22].

Subgroup analysis was also performed (G1 versus G2–G3 and G1–G2 versus G3) as endometrial cancer can be further categorized as type I (G1 and 2) and type II (G3) due to the latter being more aggressive and presenting a poorer prognosis [12,13,14]. Similarly, no statistically significant correlation was found when comparing these subgroups, as reported by Bonatti et al. [21]. However, ADC characterization of endometrial tumors is a non-consensual topic, and our findings are discrepant from those of Nougaret et al., who have previously reported lower ADC values for G3 tumors compared with G1–G2 when using total volume ADC assessment [23].

DWI/ADC assesses tissue cellularity by using the restriction of the movement of water molecules, with lower ADC values being associated with tissue having a higher cellular density [22]. The histological grade is also associated with cellular density, but there are other factors that are taken into account in DWI/ADC, such as cell proliferation, perfusion, extracellular space, and state of stroma [22], and these may act as confounders, which may justify why no statistically significant association was found between the ADC values and histological tumor grade.

DWI/ADC could be helpful also in the assessment of the tumor and in assessing the depth of myometrial invasion [21,24,25]. In our series, DCE had a higher sensitivity with similar specificity to DWI in predicting the depth of myometrial invasion, which may justify why contrast-enhanced imaging remains a recommendation in current guidelines [15]. One reason for the higher sensitivity of DCE in our cohort may be associated with the lower spatial resolution of DWI/ADC, especially when fusion software is not available. The association of T2W with DWI, in particular when fusion software is applied, results in better spatial resolution and improves the accuracy of DWI by enhancing anatomical correlation [20,24,25]. A secondary reason for the higher sensitivity of DCE in our cohort is likely linked to the DCE being acquired in multiple planes, while DWI was only acquired in the axial oblique plane, which may impair the assessment of fundal invasion. This could be overcome with the acquisition of DWI/ADC on a second orthogonal plane, such as the sagittal, improving the assessment of the uterine fundus as well as of the cervix.

The interpretation of MRI by a dedicated urogenital radiologist was found to have good agreement with histopathology (weighted kappa 0.75, *p* < 0.00) for staging endometrial cancer at our center. A similar agreement was observed by Karataşlı et al. [26] when images were read by a dedicated pelvic radiologist, with a kappa coefficient of 0.73 between MRI and histology at early stages (differentiation between stages T1a and T1b), while Bouche et al. [27] using a variety of imaging protocols from different centers and with the expertise of the reading radiologist unknown reported a poor agreement (kappa coefficient 0.12). This puts in evidence the importance of having dedicated radiologists interpreting these studies since the interpretation influences the accuracy of the endometrial cancer staging.

The depth of myometrial invasion, which differentiates between T1a and T1b endometrial stages, is a risk factor for lymph node metastases and the reason why the preoperative MRI has a role in guiding surgical protocol, namely the need for additional lymphadenectomy in the cases where deep myometrial invasion is present [11,12,26].

The sensitivity and specificity of MRI for the assessment of the depth of myometrial invasion found in our cohort was in line with that previously reported in the literature [13,26,28]. Bi et al. found in their meta-analysis that the diagnostic accuracy was highest when jointly using T2W, DCE, and DWI to identify deep myometrial invasion [29].

The identification of cervical invasion in our study population (sensitivity 42.86%, accuracy 91.11%) showed a lower sensitivity with similar accuracy to the reported by Masroor et al. (sensitivity 92.85% and accuracy 89.28%) [30], as stated above the sensitivity would likely improve if a second DWI/ADC acquisition was performed in the sagittal plane. Low sensitivity for the detection of lymph node metastases is a common issue in imaging studies, which is in line with our sensitivity of 66.67%. The reason for the low sensitivity in the identification of lymph node metastasis is related to its determination being based mainly on morphological criteria [20,31].

Although a trend towards larger lesions presenting with higher grade tumors (Table 3), also reported by Bonatti et al. [21], there was no statistically significant correlation in our cohort, which may be due to our small sample, as this association has previously been reported by Nougaret et al. [23].

Regardless of the interval between MRI and surgery (median 52 days), there was a substantial agreement between MRI and histopathological FIGO stage. There were eight cases that showed a discrepancy between MRI and surgical stage, but no statistically significant association was found in the time lag between MRI and surgery that could have been a justification for these cases (*p* = 0.61). The discordant cases were retrospectively reviewed to try to ascertain the cause of the divergence. Of the discordant cases, four cases were read at MRI as T1a (surgical staging demonstrated three cases of T1b and one of T2), three were interpreted as T1b (surgical staging demonstrated one case of T2 and one of T3), and one case was interpreted as T3 with cervical invasion and suspicious pelvic lymph nodes by morphologic criteria (surgical staging with lymphadenectomy demonstrated a T2 with no lymph node involvement), these are depicted in Table 3. The retrospective review of these cases is an important improvement tool, and in four of the eight discordant cases, we could identify the presence of pitfalls such as cornual involvement, the presence of uterine leiomyomas, and metallic artifacts from prosthetic hip leading to under- or over-staging. These are known pitfalls in the staging of endometrial cancer, as is adenomyosis or atrophic myometrium [32]. In three cases, even despite the retrospective assessment of the MRI, no changes could safely be identified that would suggest the correct histologic stage. The identification of involved metastatic lymph nodes is hampered by the primary use of morphological criteria, as shown in our case of false positive involvement that led to the incorrect upstaging of one patient.

The main limitations of our study reside in its retrospective design and in the inclusion of patients from a single center with a limited sample size, which might hamper the achievement of statistically significant results. Another limitation is associated with the calculation of ADC values using an ROI in a single representative slice instead of the whole volume of the lesion as previously used; the reasoning behind the use of this method is its easier applicability in clinical practice as it is less time-consuming even though likely less accurate than a volumetric approach.

## 5. Conclusions

No statistically significant association was demonstrated between lesion size and/or ADC values and histological grade when using a simple method for collecting the quantitative information (ROI in a single representative slice). MRI is an excellent tool for staging endometrial cancer, with a good agreement between MRI and surgical stage when interpreted by an experienced radiologist.

## Figures and Tables

**Table 1:** Characteristics of the cohort.

		Number of Patients (Total 45)
Age (years old) (median [minimum; maximum])		73 [44; 93]
Lesion size (volume in cm^2^) (median [minimum; maximum])		12.30 [0.15; 137.90]
Tumor histology n (%)	Endometrioid carcinoma	39 (86.67%)
	Serous carcinoma	5 (11.11%)
	Undifferentiated carcinoma	1 (2.22%)
Tumor grade n (%)	G1	15 (33.33%)
	G2	23 (51.11%)
	G3	7 (15.56%)
MRI FIGO stage n (%)	T1a	22 (48.89%)
	T1b	17 (37.78%)
	T2	1 (2.22%)
	T3	3 (6.67%)
	T4	2 (4.44%)
Time interval between MRI and surgery (days) (median [minimum; maximum])		52 [16; 110]
Surgical FIGO stage n (%)	pT1a	19 (42.22%)
	pT1b	17 (37.78%)
	pT2	4 (8.89%)
	pT3	3 (6.67%)
	pT4	2 (4.44%)

**Table 2:** ADC quantitative parameters and tumor grade.

		Histologic Tumor Grade	Median	Interquartile Range		Min	Max	*p*
				1Q	3Q			
Full lesion ROI	Mean ROI area (cm^2^)	G1	1.76	0.97	4.84	0,24	7,61	
		G2	2.43	1.7	5.9	0,23	12,18	0.31
		G3	6.01	2.04	7.17	0.21	12.73	
	Mean ADC (× 10^−6^ mm^²^/s)	G1	736.43	709.33	828.7	618.02	1302	
		G2	764.18	687.07	864.1	618.02	1302.78	0.99
		G3	810.60	661.18	948.14	561.42	1423.88	
	Minimum ADC (× 10^−6^ mm^²^/s)	G1	514	431	559	246	807	
		G2	522	416	598	254	818	0.90
		G3	529	268	684	198	1062	
	Maximum ADC (× 10^−6^ mm^²^/s)	G1	1098	1004	1327	929	1366	
		G2	1002	973	1199	876	1688	0.63
		G3	1167	906	1179	900	1750	
Focused ROI	Mean ROI area (cm^2^)	G1	21.97	0.07	0.14	0.05	0.24	
		G2	24.46	0.07	0.196	0.03	1.11	0.72
		G3	20.43	0.055	0.12	0.05	0.2	
	Mean ADC (× 10^−6^ mm^²^/s)	G1	24.67	576.33	741	361	839.5	
		G2	22.52	575.78	681.66	425.63	937.33	0.81
		G3	21.00	426	709	349.33	1166	
	Minimum ADC (× 10^−6^ mm^²^/s)	G1	25.00	446	639	246	694	
		G2	21.48	416	597	314	831	0.71
		G3	23.71	365	631	198	906	
	Maximum ADC (× 10^−6^ mm^²^/s)	G1	24.70	663	860	445	1094	
		G2	23.20	669	802	553	1049	0.61
		G3	18.71	547	789	434	1437	

**Table 3:** Cross table FIGO Staging MRI versus Histopathology.

FIGO Stage		Histopathology (n, %)					Total
		pT1a	pT1b	pT2	pT3	pT4	
MRI (n, %)	T1a	18 (40.00%)	3 (6.67%)	1 (2.22%)	0	0	22 (48.89%)
	T1b	1 (2.22%)	14 (31.11%)	1 (2.22%)	1 (2.22%)	0	17 (37.78%)
	T2	0	0	1 (2.22%)	0	0	1 (2.22%)
	T3	0	0	1 (2.22%)	2 (4.44%)	0	3 (6.67%)
	T4	0	0	0	0	2 (4.44%)	2 (4.44%)
Total		19 (42.22%)	17 (37.78%)	4 (8.89%)	3 (6.67%)	2 (4.44%)	45

The shaded values depict the agreement between MRI and histopathology regarding FIGO stage. Cohen’s kappa coefficient agreement yielded a good agreement (k = 0.72).

**Table 4:** Lesion volume and tumor grade.

	Histologic Tumor Grade	Median	Interquartile Range		Min	Max	*p*
			1Q	3Q			
Mean lesion volume	G1	9.87	2.46	20.22	1.75	87.92	
	G2	11.35	6.63	26.5	0.15	131.82	0.20
	G3	32.82	12.3	57.58	1.26	137.90	

## Data Availability

No new data were created or analyzed in this study. Data sharing is not applicable to this article.

## Supplementary Materials

The following supporting information can be downloaded at: https://www.mdpi.com/article/10.3390/jpm13050728/s1, Table S1: MRI scanning protocol.
