# Supplementary material for: Endometrial Cancer Staging: Is There Value in ADC?
Source: J Pers Med. 2023 Apr 25;13(5):728. doi: 10.3390/jpm13050728 (PMC10221521; doi:10.3390/jpm13050728)
Supplement: Supplementary file 1 [file jpm-13-00728-s001.zip › jpm-2327067-supplementary.pdf]

Table S1. MRI scanning protocol.

Sequence	TR/TE (ms)	Slice/Increment (mm)	Matrix	Flip angle (degrees)	FOV (mm)	b-values (s/mm2)
T2W axial pelvis	8470/99	5.0/1.0	448 × 402	150	340	
T1W axial	571/11	5.0/0.1	384 × 306	150	340	
T2W sagittal	4311/101	3.5/0.7	320 × 320	150	220	
T2W axial oblique	4160/101	3.5/0.7	320 × 320	150	220	
T2W coronal oblique	3100/101	3.5/0.3	320 × 320	150	230	
T2W abdomen	3050/125	6.0/1.8	256 × 256	150	370	
DWI	4101/84	3.5/0.7	102 × 102		220	50, 500, 1000
DCE	5.86/2.74	2.5/0.5	320 × 280	10	300	
